# Supplementary material for: Development of a CRISPR/Cas9n-based tool for metabolic engineering of Pseudomonas putida for ferulic acid-to-polyhydroxyalkanoate bioconversion
Source: Commun Biol. 2020 Mar 5;3:98. doi: 10.1038/s42003-020-0824-5 (PMC7058019; doi:10.1038/s42003-020-0824-5)
Supplement: Supplementary file 1 — Supplementary Information [file 42003_2020_824_MOESM1_ESM.pdf]

Supplementary Figures

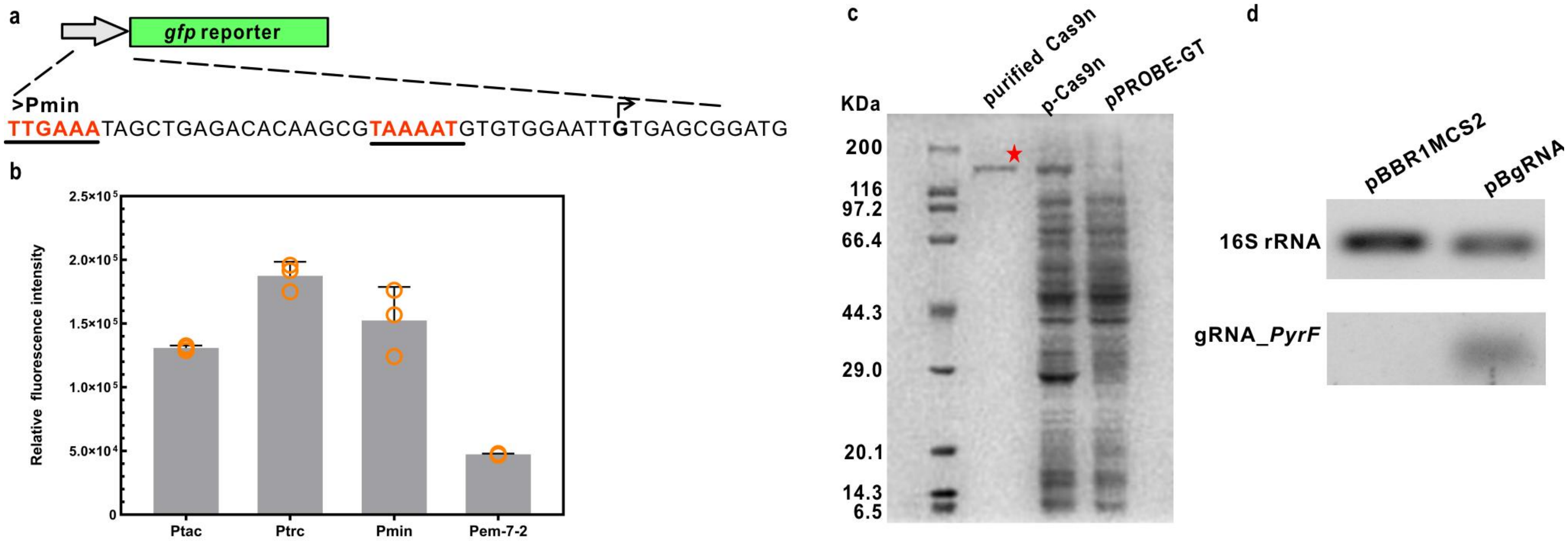

Supplementry Figure 1 Cas9n expression system in *P. putida* KT2440. **a** Synthetic promoter P_{min}[1]. Right-angled arrow indicates the potential transcriptional start site. The -35 and -10 regions are in red. **b** Promoter activity test for the four constitutive promotes. The em7-2, trc and tac promoters are referenced from previous studies [3, 6, 7]. Each promoter drives a florescent protein-encoding gene (*gfp*). Fluorescence intensity, which was detected to determine the promoter activity, is expressed in arbitrary units normalized for 10^6 CFU. **c** SDS-PAGE analysis of whole-cell proteins from transformants with control vector (pPROBE-GT) and pCas9n. The full-length His-tagged Cas9 is purified by Ni-NTA

column and denoted by the red star. **d** RT-PCR analysis of gRNA in KT2440 carrying either pBBR1MCS2 and pBgRNA, using 16S rRNA gene as an internal calibrator.

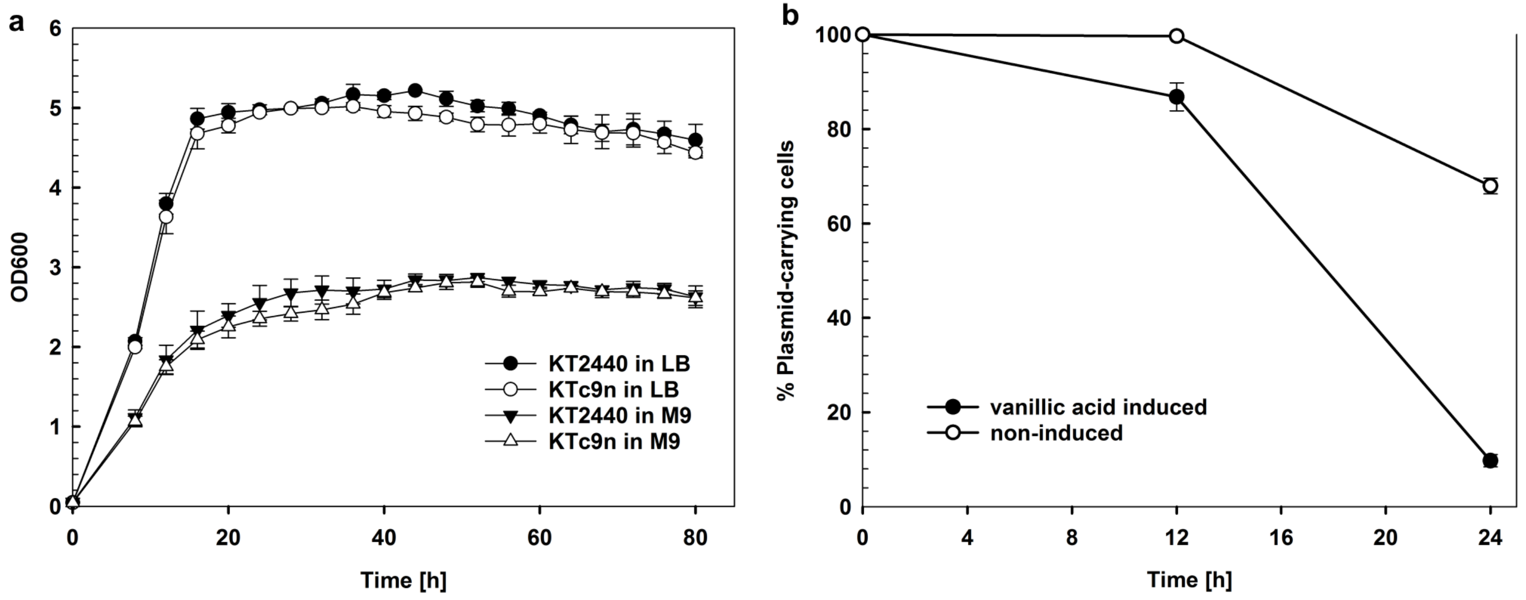

Supplementry Figure 2 Cell growth and time course characterization of the plasmid-curing system. **a** Growth curves of KT2440 and KTc9n stains in LB and 15 mM glucose-M9 minimal (supplemented with 20 μg/mL uracil) medium, respectively. **b** Curing of pBBR1-3 expressing GFP is shown as an example. The black circle indicates induced cultures with vanillic acid, whereas the white circle represents non-induced. The percentage of plasmid-carrying cells was determined by the ratio between fluorescent and the total number of cells.

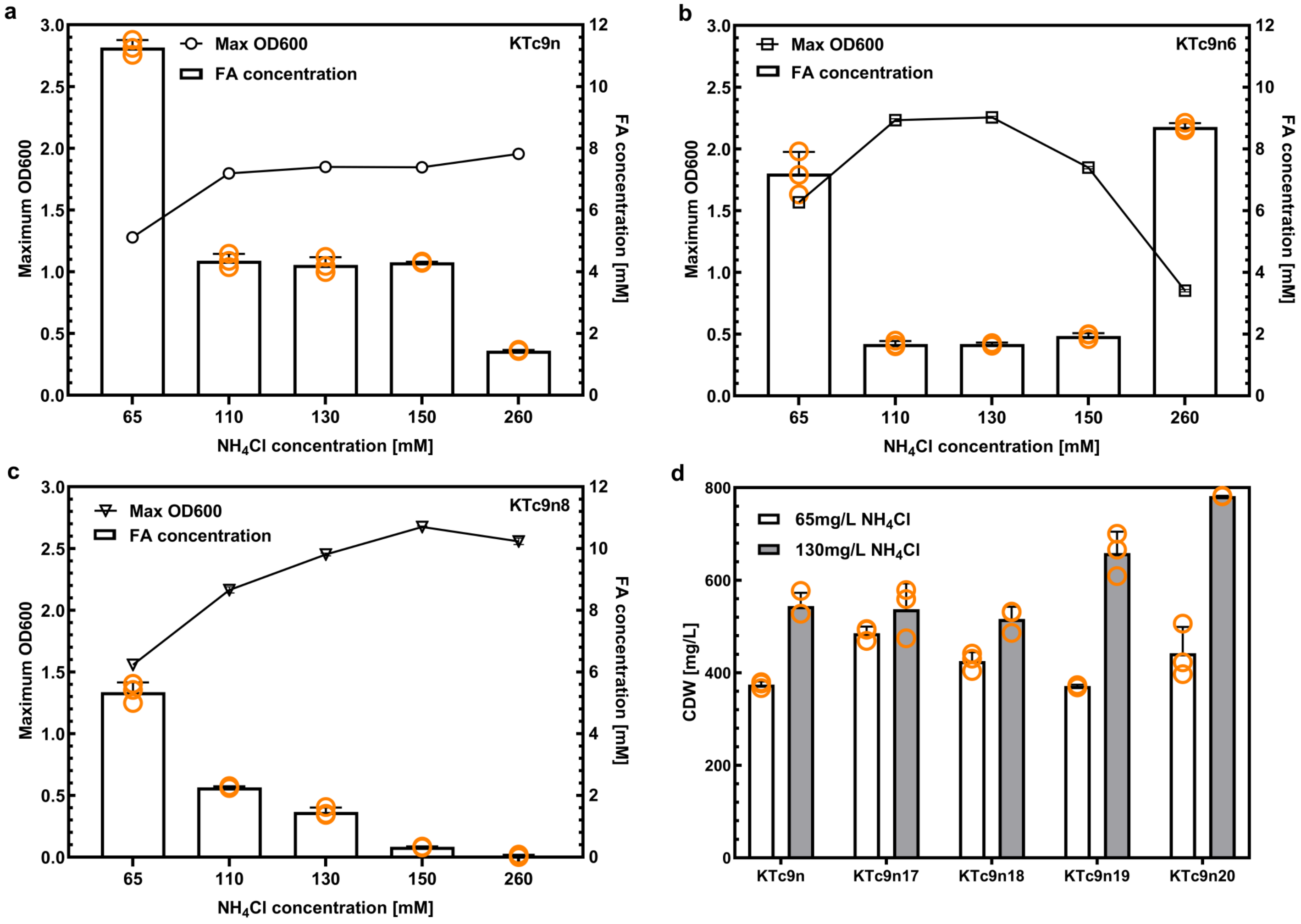
a
Max OD600
FA concentration
KTc9n
Maximum OD600
FA concentration [mM]
NH4Cl concentration [mM]
b
KTc9n6
c
KTc9n8
d
65mg/L NH4Cl
130mg/L NH4Cl
CDW [mg/L]
KTc9n
KTc9n17
KTc9n18
KTc9n19
KTc9n20

Supplementry Figure 3 Growth and ferulic aicd (FA) consumption of *P. putida* KT2440 mutants. **a-c** The maximum OD_{600} values and residual ferulic acid in medium of KTc9n, KTc9n6 and KTc9n8, when they were cultivated in 20 mM ferulic acid-M9 mineral medium, supplemented with 65-260 mg/L NH_4Cl. **d** Cell dry weights of mutants under 65 mg/L and 130 mg/L NH_4Cl conditions.

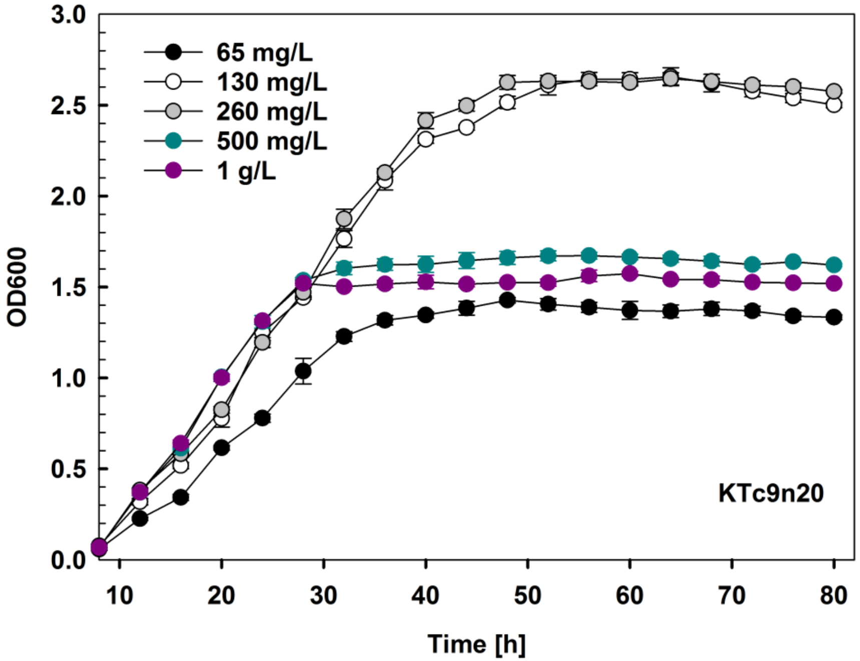

Supplementry Figure 4 Growth of KTc9n20 in 20 mM ferulic acid-M9 mineral medium, supplemented with 65-1000 mg/L NH_4Cl.

Supplementary Tables

Supplementary Table 1 Primers used in this study.

Name	Sequence 5’-3’	Application
P_{min} F	tcggGGATCCattgaaatagctgagacacaagcg	to amplify the Pmin promoter for pCas9n and pCas9 shuttle vectors
P_{min} R	gcgaccGAGCTCctcctagtgtgaaattgttatccgc	to amplify the Pmin promoter for pCas9n and pCas9 shuttle vectors
Cas9 F	ctcgatcggcctggATatcg	mutagenic primer for pCas9
Cas9 R	cgatATccaggccgatcgag	mutagenic primer for pCas9
RED F	atgaaccaaggagagtcgcaGGTACCatggatattaatactgaaactgagatcaag	to amplify the gam-bet-exo sequence
RED R	gtcgcGGATCCgctcctactggtattggcacaaacc	to amplify the gam-bet-exo sequence
P_{xylA} F	gacccAAGCTTgcacgtgggaacgatcttag	to amplify the P_{xylA} promoter
P_{xylA} R	GGTACCtgcgactctccttggttcat	to amplify the P_{xylA} promoter
P_{trc} F	ccgcGGATCCgcaacgcaattaatgtgag	to amplify the P_{trc} promoter
P_{trc}(*pyrF*)R	tgtgtgacaagggcttcgaatgtgtgaaattgttatccgct	to amplify the P_{trc} promoter and 20bp sgRNA sequence for *pyrF* gene
Scaffold(*pyrF*)F	ttcgaagcccttgtcacacagttttagagctagaaatagcaagtt	to amplify the gRNA scaffold sequence
scaffold R	caggtcgacgatactcgag	to amplify the gRNA scaffold sequence
pyrF up F1	ccggGGTACCGGATTTGGCTACAGGCACAAG	to amplify upstream region of *pyrF* gene for pDonor, pGRNA-donor, pBBR1-1
pyrF up F	ccgcGGATCCggatttggctacaggcacaag	to amplify upstream region of *pyrF* gene for pDonor, pGRNA-donor, pBBR1-1, pBBR1-2, pBBR1-2-1
pyrF up R	ctggacagccgcaggttaaatgccctggtatgcctgg	to amplify upstream region of *pyrF* gene for

		pDonor, pGRNA-donor, pBBR1-1
pyrF down F	ttaacctgcggctgtccag	to amplify downstream region of *pyrF* gene for pDonor, pGRNA-donor, pBBR1-1
pyrF down R	ccggGGTACCgtgccgagaagtgccag	to amplify downstream region of *pyrF* gene for pDonor, pGRNA-donor, pBBR1-1, pBBR1-2
pyrF down R2	cccgGAATTCgtgccgagaagtgccagta	to amplify downstream region of *pyrF* gene for pDonor, pGRNA-donor, pBBR1-1
rrnB R	ccggGGTACCaaaaggccatccgtcaggat	to amplify rrnB transcription terminator T1T2 for pgRNA, pBgRNA, pBBR1-1, pGRNA-donor
rrnB R2	ccgcGGATCCaaaaggccatccgtcaggat	to amplify rrnB transcription terminator T1T2 for pBBR1-1
pUR	cccAAGCTTatgccctggtatgcctgg	to amplify upstream region of *pyrF* gene for pBBR1-2, pBBR1-2-1
pDF	ccgGAATTCttaacctgcggctgtccag	to amplify downstream region of *pyrF* gene for pBBR1-2, pBBR1-2-1
sgRNA(*icd*)R	ccttcgacaccggcttgacgtgtgtgaaattgttatccgct	to amplify the Ptrc promoter and 20bp sgRNA sequence for *icd* gene
scaffold(*icd*)F	cgtcaagccggtgtcgaagggttttagagctagaaatagcaagtt	to amplify the gRNA scaffold and 20bp sgRNA sequence for *icd* gene
rrnB F1	ctcgagtatcgtcgacctgcaaataaaacgaaaggctcagtcg	to amplify the terminator rrnB T1T2 for pBBR1-3, pgRNA, pBgRNA, pBBR1-1, pGRNA-donor
rrnBT7 R2	CCCCTTGGGGCCTCTAAACGGGTCTTGAGGGGTTTTTTGGGTACCaaaaggccatccgt	the terminator rrnB T1T2 for pBBR1-3
rrnBT7 R3	cagtccagagaaatcggCTAGCATAACCCCTTGGGGCCTCTAAAC	to amplify the terminator T7 for pBBR1-3
rrnBT7 R4	gccgGAATTCAAGCTTcatggccacgcagtccagagaaatcggCTAG	to amplify the terminator T7 and palindromic

		sequence for pBBR1-3
P_{van} F	acgggaattccatggccacgcagtccagagaaatcggCCAGGAATTGGGGATCGGA	to amplify the P_{van} promoter and palindromic sequence for pBBR1-3
P_{van} R	tctctggactgcgtggccatgcttggatccattaaattggagagtcagg	to amplify the P_{van} promoter and 20bp sgRNA sequence guiding palindromic sequence for pBBR1-3
scaffold F2	catggccacgcagtccagagaaatgttttagagctagaaatagcaagtt	to amplify the gRNA scaffold and 20bp sgRNA sequence guiding palindromic sequence for pBBR1-3
scaf t500 R	ggcctttTacaaagcccgccgaaaggcgggcttttctgtcaggtcgacgatactcgag	to amplify the gRNA scaffold and t500 terminator for pBBR1-3
t500tR2 R	ctagTCTAGAaacaggcctgctggtaatcgcaggcctttTacaaagcccgc	to amplify the tR2 terminator for pBBR1-3
icd up F	ctagTCTAGAgcggatgaagtggatgatactc	to amplify upstream region of *icd* gene for pBBR1-3, pBBR1-4
icd up R	cccgatctaaggagaacgacaatcctctcggcagagatgatg	to amplify upstream region of *icd* gene for pBBR1-3
icd pyrF F	catcatctctgccgagaggattgtcgttctccttagatcggg	to amplify *pyrF* gene for pBBR1-3
pyrF F	tgtcgttctccttagatcggg	to amplify *pyrF* gene for pBBR1-3 KTc9nΔvanAB
pyrF R	ttacccacggatctccgcc	to amplify *pyrF* gene for pBBR1-3
icd down F	tggcggagatccgtgggtaaacgttaagcgaatggtctg	to amplify downstream region of *icd* gene for pBBR1-3
icd down R	gcccAAGCTTgaattctcgtcggtgaaggtatagatgat	to amplify downstream region of *icd* gene for pBBR1-3, pBBR1-4
P_{tac} F	accgGAATTCAAGCTTacgttatcgactgcacgg	to amplify the P_{tac} promoter for pBBR1-3
P_{tac} R	gcgaccgagctcctccttgtgtgaaattgttatccg	to amplify the P_{tac} promoter for pBBR1-3

gfp F	cacaaggaggagctcggtcgcatgagtaaaggagaagaacttttcact	to amplify *gfp* gene for pBBR1-3
gfp R	ccggGGTACCctatttgtatagttcatccatgccat	to amplify *gfp* gene for pBBR1-3
icd down F2	catcatctctgccgagaggatacgttaagcgaatggtctg	to amplify downstream region of *icd* gene for pBBR1-4
icd up R2	atcctctcggcagagatgatg	to amplify downstream region of *icd* gene for pBBR1-4
P_{trc}(*tesA*)R	ggcttggtttatgtgctgcatgtgtgaaattgttatccgct	to amplify the Ptrc promoter and 20bp sgRNA sequence guiding *tesA* gene for pBBR1-5
scaffold(*tesA*) F	tgcagcacataaaccaagccgttttagagctagaaatagcaagtt	to amplify the gRNA scaffold and 20bp sgRNA sequence guiding *tesA* gene for pBBR1-5
tesA up F	ctagTCTAGAggtgtggctgtcgaggtta	to amplify upstream region of *tesA* gene for pBBR1-5, pBBR1-6
tesA up R	cccgatctaaggagaacgacacgctggtatcgccactgat	to amplify upstream region of *tesA* gene for pBBR1-5
tesA down F	ggcggagatccgtgggtaacccgcacaattgcaacaaa	to amplify downstream region of *tesA* gene for pBBR1-5
tesA down R	tcgataacgtaagcttattccccgaaaggtgggtcaagagga	to amplify downstream region of *tesA* gene for pBBR1-5, pBBR1-6
tesA down F2	atcagtggcgataccagcgcccgcacaattgcaacaaa	to amplify upstream region of *tesA* gene for pBBR1-6
tesA up R2	tttgttgcaattgtgcgggcgctggtatcgccactgat	to amplify downstream region of *tesA* gene for pBBR1-6
P_{trc} (*tesB*)R	cgatggattcgaggctcaactgtgtgaaattgttatccgct	to amplify the Ptrc promoter and 20bp sgRNA sequence guiding *tesB* gene for pBBR1-7
sg *tesB* F	gttgagcctcgaatccatcggttttagagctagaaatagcaagtt	to amplify the gRNA scaffold and 20bp sgRNA sequence guiding *tesB* gene for pBBR1-7

tesB up F	ctagTCTAGAccttgcgatggcgaatcag	to amplify upstream region of *tesB* gene for pBBR1-7, pBBR1-8
tesB up R	cccgatctaaggagaacgacacattcgttgcacggctactt	to amplify upstream region of *tesB* gene for pBBR1-7
tesB down F	ggcggagatccgtgggtaatgactcatcggcggttctc	to amplify downstream region of *tesB* gene for pBBR1-7
tesB down R	GCCCaagcttccaatggcatgacgatgactt	to amplify downstream region of *tesB* gene for pBBR1-7, pBBR1-8
tesB up R2	cattcgttgcacggctactt	to amplify upstream region of *tesB* gene for pBBR1-8
tesB down F2	aagtagccgtgcaacgaatgtgactcatcggcggttctc	To amplify downstream region of *tesB* gene for pBBR1-8
P_{trc} (*phaC1*)R	ggccgtagtcgaggtagtcgtgtgtgaaattgttatccgct	to amplify the Ptrc promoter and 20bp sgRNA sequence guiding *phaC1* gene for pBBR1-9
sg *phaC1* F	cgactacctcgactacggccgttttagagctagaaatagcaagtt	to amplify the gRNA scaffold and 20bp sgRNA sequence guiding *phaC1* gene for pBBR1-9
phac1 up F	ctagtctagagctggcacagaacgaagac	to amplify upstream region of *phaC1* gene for pBBR1-9, pBBR1-10, pBBR1-11
phac1 up R	cccgatctaaggagaacgacattgtcctgagacgagtaccg	to amplify upstream region of *phaC1* gene for pBBR1-9
phac1 down F	tggcggagatccgtgggtaacagacgagggagtgttgcc	to amplify downstream region of *phaC1* gene for pBBR1-9
phac1 down R	gcccaagcttcgctgaggtcgaagatgtaga	to amplify downstream region of *phaC1* gene for pBBR1-9, pBBR1-10, pBBR1-11
*xho*I C11R	cccgCTCGAGtcaacgctcgtgaacgtagg	to amplify upstream region and a copy *phaC1*(*TphaC1*) gene or for pBBR1-10

		(pBBR1-11)
*xho*I C11F	cccgCTCGAGaacggagcgtcgtagatgag	to amplify downstream region and a copy *phaC1* gene or for pBBR1-10
*phaC*1 F kpnI	acggGGTACCGGCCTGCGGGGTTTAGAG	to amplify downstream region and a copy *TphaC1* gene or for pBBR1-10
T*phaC1* GF	ggcgggcgtgctgggcggca	mutagenic primer for *TphaC1*
T*phac1*G R	tgccgcccagcacgcccgcc	mutagenic primer for *TphaC1*
T*phaC1* SF	cgacatcctgttctggTCGaa	mutagenic primer for *TphaC1*
T*phaC1* SR	ttCGAccagaacaggatgtcg	mutagenic primer for *TphaC1*
SR2	ggaaggcggccggcaggcgcgtggtgtcgttCGAccagaacaggatgtcg	mutagenic primer for *TphaC1*
SF2	ctggTCGaacgacaccacgcgcctgccggccgccttccacggcgacctgatcg	mutagenic primer for *TphaC1*
SF3	ccttccacggcgacctgatcg	mutagenic primer for *TphaC1*
P_{trc} (*fcs*)R	gtgaagttgacccggcgtactgtgtgaaattgttatccgct	to amplify the Ptrc promoter and 20bp sgRNA sequence guiding *ech-vdh-fcs* genes for pBBR1-12
sg *fcs* F	gtacgccgggtcaacttcacgttttagagctagaaatagcaagtt	to amplify the gRNA scaffold and 20bp sgRNA sequence guiding *ech-vdh-fcs* genes for pBBR1-12
fcs up F	ctagtctagagagcttgtccacatcgagttc	to amplify upstream region of *ech-vdh-fcs* genes for pBBR1-12; To amplify upstream region and a copy *ech-vdh-fcs* genes for pBBR1-13, pBBR1-14, pBBR1-15
fcs up R	cccgatctaaggagaacgacaccatcgacctgggcattca	to amplify upstream region of *ech-vdh-fcs* genes for pBBR1-12
fes down F	tggcggagatccgtgggtaatagtaccgacgacgcttgc	to amplify downstream region of *ech-vdh-fcs* genes for pBBR1-12

fes down R	cgataacgtaagcttattcccacaggtcgccaaggatattct	to amplify downstream region of *ech-vdh-fcs* genes for pBBR1-12; To amplify downstream region and the second copy of *ech-vdh-fcs* genes for pBBR1-13
3359 up R	cccgGAATTCTGACGAAAGGACTGAACTGTTG	to amplify downstream region of *ech-vdh-fcs* genes for pBBR1-14
3359 up R smaI	atccCCCGGGTGACGAAAGGACTGAACTGTTG	to amplify downstream region of *ech-vdh-fcs* genes for pBBR1-15
2 *fes* up R	gccgGAATTCTGCTCCTCGATCATGGGTAAT	to amplify upstream region and a copy *ech-vdh-fcs* genes for pBBR1-13, pBBR1-14, pBBR1-15
2 *fes* down F	tccgGAATTCtgtcctcgaagacggtgc	to amplify downstream region and the second copy of *ech-vdh-fcs* genes for pBBR1-13, pBBR1-15
3359 down R	cgataacgtaagcttattcccacaggtcgccaaggatattct	to amplify downstream region of *ech-vdh-fcs* genes for pBBR1-14， pBBR1-15
Ptrc (*vanAB*)R	cattgatcgatctcgccggttgtgtgaaattgttatccgct	to amplify the Ptrc promoter and 20bp sgRNA sequence guiding *vanAB* gene for pBBR1-16
sg *vanAB* F	accggcgagatcgatcaatggttttagagctagaaatagcaagtt	to amplify the gRNA scaffold and 20bp sgRNA sequence guiding *vanAB* gene for pBBR1-16
vanAB up F	ctagTCTAGAGCCAGTGAAGATGCTGAAGC	to amplify upstream region of *vanAB* gene for pBBR1-16, pBBR1-17 , pBBR1-18
vanAB up R2	cccgatctaaggagaacgacagccgaacctgctatgccta	to amplify upstream region of *vanAB* gene for pBBR1-16
vanAB down F2	ggcggagatccgtgggtaagaggcgaatggcaggtgat	to amplify downstream region of *vanAB* gene for pBBR1-16

vanAB down R	accgtgcagtcgataacgtaagcttattcccgcagcaatggcaaggagtgg	to amplify downstream region of *vanAB* gene for pBBR1-16, pBBR1-17 , pBBR1-18
vanB R2	ccggGGTACCgtcagatgtccagcaccagc	to amplify upstream region and a copy *vanAB* gene for pBBR1-17
van F kpnI	ccggGGTACCTTGTAGAGTTGTTCGATGGCTAG	to amplify downstream region and a copy *vanAB* gene for pBBR1-17
vanB EcoRI	cccgGAATTCtcagatgtccagcaccagc	to amplify a copy *vanAB* gene for pBBR1-18
van F EcoRIGA	CCCGgaattcTTGTAGAGTTGTTCGATGGCTAG	to amplify downstream region and a copy *vanAB* gene for pBBR1-18
P_{trc} (*tesBII*) R	ATCCGCTTCGAGGTGGAGGTtgtgtgaaattgttatccgct	to amplify the P_{trc} promoter and 20bp sgRNA sequence guiding *tesB II* gene for pBBR1-19
sg *tesB II* F	ACCTCCACCTCGAAGCGGATgttttagagctagaaatagcaagtt	To amplify the gRNA scaffold and 20bp sgRNA sequence guiding *tesB II* gene for pBBR1-19
tesB II up F	ctagTCTAGAGCGGTTCTACATTCGTGAATCT	to amplify upstream region of *tesB II* gene for pBBR1-19, pBBR1-20
tesB up R2	cccgatctaaggagaacgacagcggaatgctgacggaatc	to amplify upstream region of *tesB II* gene for pBBR1-19
tesB II down F2	ggcggagatccgtgggtaagtggtggaagaacaggtgaac	to amplify downstream region of *tesB II* gene for pBBR1-19
tesB II down R	ccgtgcagtcgataacgtaagctttgatcgctgtgccgttgt	to amplify downstream region of *tesB II* gene for pBBR1-19, pBBR1-20
tesB II up R3	gttcacctgttcttccaccacgcggaatgctgacggaatc	to amplify upstream region of *tesB II* gene for pBBR1-20
tesB II down F3	gattccgtcagcattccgcgtggtggaagaacaggtgaac	to amplify downstream region of *tesB II* gene for pBBR1-20
pyrF seq F	gcacggcagtgatcctctt	to sequence KTΔ*pyrF*

pyrF seq F	gaacgaaggcaagtcctacatc	to sequence KTΔ*pyrF*
cas9-7	ggctacaaggaagtgaagaagg	to sequence KTc9n
icd seq R	gatgttcggcagcttgatgat	to sequence KTc9n▵*icd*
fcs seq F	ctgccgaacagcatctcttg	to sequence KTc9n1 KTc9n2 KTc9n3 KTc9n7 KTc9n8
fcs seq R	aagttgatcgcctgctgaatg	to sequence KTc9n1 KTc9n2 KTc9n3 KTc9n7 KTc9n8 KTc9nΔ*fcs-ech-vdh* KTc9n1 *fcs-ech-vdh*
vanAB seq F	gtgttcagttgacgcaaggt	to sequence KTc9n4 KTc9n5 KTc9n6 KTc9n7 KTc9n8
vanAB seq R	ggtgaggtccatgaggttgt	to sequence KTc9n4 KTc9n5 KTc9n6 KTc9n7 KTc9n8 KTc9nΔ*vanAB*
tesA seq F	ggtgtggctgtcgaggtta	to sequence KTc9n10
tesA seq R	gccaggttgatgacgatgc	to sequence KTc9n10
tesB seq F	ccttgcgatggcgaatcag	to sequence KTc9n11 KTc9n15 KTc9n16 KTc9n17 KTc9n18 KTc9n19 KTc9n20
tesB seq R	gtcactcagttgttgctcgtt	to sequence KTc9n11 KTc9n15 KTc9n16 KTc9n17 KTc9n18 KTc9n19 KTc9n20
tesB IIseq F	tcggatgtgacgctggatg	to sequence KTc9n12 KTc9n15 KTc9n16 KTc9n17 KTc9n18 KTc9n19 KTc9n20
tesB IIseq R	acagtattgccgaacaga	to sequence KTc9n12 KTc9n15 KTc9n16 KTc9n17 KTc9n18 KTc9n19 KTc9n20
phaC1 seq F	gcaccgctgttccttatcac	to sequence KTc9n13 KTc9n14 KTc9n16 KTc9n18 KTc9n20
phaC1 seq R	gtccagcaagctgaccaga	to sequence KTc9n13 KTc9n14 KTc9n16

GFP-R2	ccggGGTACCctatttgtatagttcatccatgccat	KTc9n18 KTc9n20 to sequence KTpGT

Supplementary Table 2 Mutation efficiency of the CRISPR/Cas9n-λ-Red system at different xylose inducer concentrations.

Target gene	System	Plasmid	Component	The concentration of xylose （mM）	Efficiency
pyrF	CRISPR/Cas9n-λ Red	pCas9n-Red, pBBR1-1	λ-Red recombinase<br>Cas9n<br>gRNA<br>homologous arms	2	1/ 2146
				4	1/108
				8	1/171
				16	1/10

Supplementary Table 3 Application of CRP genome editing system in *P. putida* KT2440.

**Location (GenBank: AE015451.2)**	**Deletion**	**Insertion**	**Size (bp)**	**Mutation efficiency (mutants/colonies)**	**Mutant**
2040441-2041327	*pyrF* (PP_1815)	P_{min}::*cas9n*<br>P_{xylA}:: *gam-bet-exo*	885 (deletion)<br>7738 (insertion)	20/20	KTc9n
4520663-4521664	*icd* (PP_4011)	\	1000	15/15	KTc9n9
2648226-2648435	*tesA* (PP_2318)	\	98	8/8	KTc9n10
5422549-5422711	*tesB* (PP_4762)	\	163	16/16	KTc9n11
37799087-3799516	*ferR* (PP_3359)	\	428	8/8	KTc9n3
3799087-3799516	*ferR* (PP_3359)	*fcs-ech-vdh*	428 (deletion)<br>4617 (insertion)	8/8	KTc9n4
2637955-2638411	*tesBII* (PP_2308)	\	455	16/16	KTc9n12
4263475-4265971	*vanAB* (PP_3736-3737)	\	2495	8/8	KTc9nΔ*vanAB*
3794470-3799114	*fcs-ech-vdh* (PP_3356- 3358)	\	4643	8/8	KTc9nΔ*fcs-ech-vdh*
5422549-5422711<br>2637955-2638411	*tesB* (PP_2308)<br>*tesBII* (PP_4762)	\	163 (*tesB*)<br>455 (*tesB*II)	20/20 for *tesB*<br>20/20 for *tesBII*	KTc9n15
5699249-5699250	\	mutated *phaC1*	1959	8/8	KTc9n14
4265970-4265971	\	*vanAB*	2459	8/8	KTc9n5
3794470-3794471	\	*fcs-ech-vdh*	4617	8/8	KTc9n1

3794470-3794471<br>4265970-4265971	\	*fcs-ech-vdh*<br>*vanAB* gene	4617<br>2459	8/8	KTc9n6
3799087-3799516<br><br>4265970-4265971	*ferR* (PP_3359)	*fcs-ech-vdh*<br><br>*vanAB*	428 (deletion)<br>4617 (insertion)<br>2459 (insertion)	8/8	KTc9n7
3799087-3799516<br>4265970-4265971	*ferR* (PP_3359)	*fcs-ech-vdh*<br>two copies of *vanAB*	428 (deletion)<br>4617 (insertion)<br>4918 (insertion)	8/8	KTc9n8
3794470-3794471	\	two copies of *fcs-ech-vdh*	9124	20/20	KTc9n2
2040441-2041327	\	pPROBE-GT DNA sequence	15000	15/15	KTpGT
5701202-5702147 (deletion)<br>5701202-5701203 (insertion)	*phaZ* (PP_5004)	*phaC1* (PP_5003)	944 (deletion)<br>1701 (insertion)	12/12	KTc9n13

Supplementary Table 4 *mcl*-PHA composition of the engineered *P. putida* KT2440 strains.

Strain	Concentration of ferulic acid (mM)	Concentration of NH_4Cl (mg/L)	PHA composition (mol%)[a]			
			3HHx (C_6)	3HO (C_8)	3HD (C_{10})	3HDD (C_{12})
KT2440	20	65	4.80±0.36	28.07±2.27	60.94±3.09	6.19±0.46
KTc9n	20	65	4.21±0.71	25.88±2.57	64.82±4.63	5.09±1.36
KTc9n1	20	65	4.09±0.19	23.47±0.44	67.38±0.59	5.06±0.19
KTc9n6	20	65	3.63±0.19	21.24±0.92	69.30±1.18	5.83±0.15
KTc9n8	20	65	3.81±0.03	23.21±0.93	67.44±0.79	5.54±0.11
KTc9n9	20	65	3.77±0.17	21.07±0.70	69.72±0.48	5.44±0.25
KTc9n10	20	65	3.74±0.12	21.64±0.06	69.12±0.09	5.50±0.12
KTc9n11	20	65	3.66±0.07	21.90±0.13	68.96±0.12	5.48±0.04
KTc9n12	20	65	2.93±0.45	20.29±1.04	70.62±1.15	6.16±0.34
KTc9n13	20	65	3.71±0.07	22.68±0.35	68.55±0.50	5.06±0.28
KTc9n14	20	65	3.49±0.03	22.13±0.16	68.21±0.16	6.17±0.01
KTc9n15	20	65	3.46±0.67	21.28±1.30	69.37±1.71	5.89±0.26
KTc9n16	20	65	3.31±0.02	22.07±0.17	68.50±0.23	6.12±0.10
KTc9n17	20	65	3.74±0.05	21.67±0.24	68.85±0.25	5.74±0.07
KTc9n18	20	65	3.23±0.10	21.58±0.43	68.79±0.45	6.40±0.07
KTc9n19	20	65	3.93±0.11	22.49±0.45	68.24±0.19	5.34±0.47
KTc9n20	20	65	3.90±0.13	22.40±0.57	67.90±0.28	5.80±0.87
KTc9n	20	130	3.33±0.56	21.30±1.44	69.63±1.89	5.74±0.12
KTc9n17	20	130	3.42±0.20	23.75±1.12	67.66±0.77	5.17±0.18
KTc9n18	20	130	3.30±0.03	23.07±0.50	67.72±0.42	5.91±0.13
KTc9n19	20	130	3.71±0.15	23.50±0.89	66.68±1.29	6.11±0.26

KTc9n20	20	130	3.88 ±0.04	24.29 ±0.50	65.59 ±0.74	6.24 ±0.28

[a]3HHx, 3-hydroxyhexanoate; 3HO, 3-hydroxyoctanoate; 3HD, 3-hydroxydecanoate; 3HDD, 3-hydroxydodecanoate

Supplementary Notes

Deletion of *pyrF* by the CRISPR/Cas9-λ-Red system.

To investigate the system, the **pCas9** plasmid was first constructed, which expresses Cas9 protein under the control of the constitutive P_{min} promoter [42]. Subsequently, the λ-Red expression cassette (P_{xylA}::*gam-bet-exo*) was introduced into **pCas9** to improve repairing efficiency and thus alleviate the toxicity of Cas9 caused DSB. It generated the construct pCas9-Red (Table 1). Meanwhile, the other vector, **pBBR1-1** [43], was constructed to express the customized sgRNA for *pyrF* under control of the P_{trc} promoter and contain 0.5 kb homologous repairing arms, providing homology-directed repair to fix DNA lesions (Table 1). The **pCas9** and **pBBR1-1** were co-transformed into KT2440. Consistent with previous reports [30, 41, 44], the co-expression vectors produced cells with neither antibiotic resistance nor 5-FOA resistance, whereas two controls (KT2440 carrying either **pCas9** or **pBBR1-1**) generated antibiotic-resistant transformants (Table 2). Together, this suggested Cas9-induced chromosomal cleavage is indeed toxic in *P. putida* KT2440, at least at the selected gene locus. In contrast, when **pCas9-Red** and **pBBR1-1** were co-transformed into KT2440, dozens of colonies were observed on 5-FOA+Gm^r+Km^r LB selection plates. This indicated *pyrF* was knocked-out, and was further confirmed by colony PCR screening and DNA sequencing. Hence, this CRISPR/Cas9-λ-Red system works in KT2440, consistent with the previous study [30].

Effect of isocitrate dehydrogenase deletion on *mcl*-PHA biosynthesis.

mcl-PHA is produced from fatty acid biosynthesis pathway, when *P. putida* utilizes lignin-derived substrates (non-fatty acid feedstock) as carbon sources [48] (Figure 4a). Under this condition, acetyl-CoA is a key intermediate of the PHA biosynthesis pathway, as it plays an essential role in replenishing both the citric acid cycle and the fatty acid synthesis pathway (Figure 4a-b). Previous studies reported that inactivation of isocitrate dehydrogenase (*icd*) reduced the intracellular concentration of oxaloacetate, and consequently increased the flux of acetyl-CoA into the fatty acid biosynthesis pathway [49]. The *icd* gene was therefore knocked-out in KTc9n, generating the mutant KTc9n9 (Table 1 and Supplementary Table 3). However, the expected positive effect was not observed. It produced slightly less *mcl*-PHA than KTc9n, even though cell growth for these two strains were similar (Figure 4c-d and Supplementary Table 4). This revealed isocitrate dehydrogenase did not contribute to *mcl*-PHA synthesis in *P. putida* KT2440 under FA substrate conditions, even though it was proposed to be one of the genetic engineering targets towards improved PHA production via omics approaches [50].

Previous transcriptomic study revealed that *icd* was significantly downregulated under nitrogen limitation (PHA accumulation condition) [46], and deletion of *icd* was validated to stimulate the *mcl*-PHA biosynthesis via the fatty acid synthesis pathway in *P. putida* KT2442 with gluconate as carbon source [45]. However, in this study, inactivation of *icd* did not enhance the PHA titer when FA was utilized as the carbon source under nitrogen limiting condition, indicating the effect is variable either in different strains or under different growth conditions.

The three components in one plasmid are incompatible in *P. putida.*

Three components, *cas9n*, sgRNA, and homologous repairing arms were

introduced into the plasmid, **pPROBE-GT**, generating an all-in-one **pCas9n1** (Table 1). Transformation and three serial transfers test showed that **pCas9n1** wasn't stable in *P. putida*, although the controls **pROBE-GT**, **pCas9n**, **pgRNA**, **pDonor**, and **pgRNA-donor**, which carry none, one, or two of the three components can stably replicate in *P. putida* (Table 1). It indicated that, unlike *E.coli* and *Clostridium cellulolyticum* [34, 41], the three components in one plasmid are incompatible in *P. putida* to perform genome editing.

Supplementary Methods.

Bioinformatic analysis of target N20NGG sites.

All N20NGG sites near the target genes were extracted from both strands. Next, ideal sgRNAs were selected to meet the following criteria: (i) a string of five or less T's in the 23-mer sequence, (ii) two or less T in the 6-mer region upstream of NGG, (iii) appropriate GC content (30%-75%) and (iv) unique in the genome if up to two base-pair mismatches were allowed.

Plasmid construction.

The synthetic codon adapted *cas9n* gene was mutated from *cas9* via site-directed mutagenesis using mutatgenic primers Cas9F and Cas9R. The adapted *cas9n* (or *cas9*) fragment and the P_{min} promoter DNA fragment, amplified by PCR from pGP_{min}[1], were ligated into the plasmid **pPROBE-GT** (empty vector) to generate **pCas9n** and **pCas9** plasmids, respectively. In addition, the *gam-bet-exo* genes were amplified from lambda phage DNA (TakaRa) with primers RedF and RedR, and fused with P_{xylA} promoter DNA fragment via gene splicing by overlap extension PCR [2]. The generated P_{xylA}:: *gam-bet-exo* expression cassette was ligated into plasmids **pCas9n** and **pCas9** through *Hind*III and *BamH*I, to construct the **pCas9n-Red** and **pCas9-Red** plasmid, respectively. The **pBBR1-1** plasmid was constructed by inserting the P_{trc}:: *pyrF* gRNA

cassette and 0.5 kb *pyrF* homologous arms into **pBBR1MCS2** which was cut with *BamH*I and *Kpn*I. The *pyrF* gRNA cassette was amplified and fused with the RBS-free P_{trc} promoter that was amplified from **pTrc99A** [3]. 0.5 kb length upstream and downstream of *pyrF* homologous repairing arms were amplified from the genome of KT2440 using primers pyrFupF/pyrFupR and pyrFdownF/ pyrFdownR, respectively, and subsequently were spliced to produce the 1-kb donor. Next, the P_{trc}:: *pyrF* gRNA and 1-kb *pyrF* donor were inserted into pPROBE-GT which was cut with *BamH*I and *Kpn*I to yield the plasmid pGRNA-donor, while each of the two fragments was ligated to **pPROBE-GT** to construct the plasmids **pgRNA** and **pDonor**, respectively, as the control. Similarly, the two fragments were also ligated into **pCas9n**, digested by *HindII*I and *BamH*I, to construct **pCas9n1** (Table 1).

To integrate *cas9n* and Red expression cassettes and replace the *pyrF* gene in *P. putida* KT2440 strain, **pBBR1-2** was constructed through ligation of a 1 kb DNA fragment containing the upstream and downstream of *pyrF* homologous repairing arms, P_{min}:: *cas9n* as well as P_{xylA}:: *gam-bet-exo* expression cassettes to **pBBR1MCS2**. To inactivate the *icd* gene, plasmid **pBBR1-3** was constructed, which contains the P_{trc}:: *icd* sgRNA, a *pyrF* gene flanked by 0.5 kb *icd* homologous arms, a 66 bp palindromic sequence, a P_{tac}::*gfp* expression cassette and a P_{van}::sgRNAps expression cassette [4] that guides the palindromic sequence in the plasmids for plasmid curing. In addition, **pBBR1-4**, harboring P_{trc}:: gRNA for *pyrF*, 0.5 kb homologous arms for *icd*, P_{tac}::*gfp*, P_{van}:: sgRNAps and palindromic sequence, was constructed to remove the selection marker *pyrF* in the KTc9nΔ*icd* strain. Similarly, **pBBR1-5** and **pBBR1-6** to delete *tesA* gene, **pBBR1-7** and **pBBR1-8** to delete *tesB* gene, **pBBR1-9**, **pBBR1-10** and **pBBR1-11** to delete *phaZ*, insert *phaC1* gene, and integrate mutated *phaC1* [5], **pBBR1-2-1** and **pBBR1-2-2** to insert **pPROBE-GT** vector sequence,

pBBR1-12, **pBBR1-13** and **pBBR1-13-2** to insert either one or two copies of *ech-vdh-fcs* genes, **pBBR1-12**, **pBBR1-14** and **pBBR1-15** to delete *ferR* and insert *ech-vdh-fcs* genes, **pBBR1-16**, **pBBR1-17** and **pBBR1-18** to optimize the gene copy ratio of *ech-vdh-fcs* and *vanAB*, as well as **pBBR1-19** and **pBBR1-20** to remove *tesBII* gene were all constructed via the standard molecular cloning protocols (Table 1).

CRISPR/Cas9(n) based genome editing procedures

For the two-plasmid CRISPR/Cas9 system, the pCas9 and pBBR1-1 plasmids were co-transformed into KT2440 to delete the *pyrF* gene (Table 1). After 18 h of incubation at 30 ℃ on LB medium (Ura^++Km+Gm), appropriately diluted cells were subsequently plated on LB selective medium (Ura^++5-FOA+Km+Gm). Meanwhile. either **pCas9** or **pBBR1-1** plasmid was transformed into KT2440 as a control. Cells were then plated on LB selective medium (Ura^++5-FOA) with corresponding antibiotic (Km or Gm).

For the two-plasmid CRISPR/Cas9-λ-Red system, the **pCas9-Red** and **pBBR1-1** were co-transformed into KT2440 via electroporation. The strain was incubated at 30 ℃ in LB medium (Ura^++Km+Gm) for 18 h. 4 mM xylose was added to LB medium to induce the expression of λ-Red recombinase. Next, appropriately diluted cells were plated on LB selective agar plate (Ura+5-FOA+Km+Gm) to select the mutant strain.

Similarly, the two plasmid CRISPR/Cas9n system and CRISPR/Cas9n-λ-Red system were also tested according to the above described genome editing procedures (Table 2).

References

1. Lin L, Wang X, Cao L, Xu M. Lignin catabolic pathways reveal unique characteristics of Dye-decolorizing peroxidases in *Pseudomonas putida*. *Environ Microbiol*, (2019).
2. Wang X, *et al.* Simultaneous improvements of *Pseudomonas* cell growth and polyhydroxyalkanoate production from a lignin derivative for lignin-consolidated bioprocessing. *Appl Environ Microbiol* **84**, 01469-01418 (2018).
3. Wang Q, Tappel RC, Zhu C, Nomura CT. Development of a new strategy for production of medium-chain-length polyhydroxyalkanoates by recombinant *Escherichia coli* via inexpensive non-fatty acid feedstocks. *Appl Environ Microbiol* **78**, 519-527 (2012).
4. Lin L, *et al.* Systems biology-guided biodesign of consolidated lignin conversion. *Green Chem* **18**, 5536-5547 (2016).
5. Hiroe A, Watanabe S, Kobayashi M, Nomura CT, Tsuge T. Increased synthesis of poly(3-hydroxydodecanoate) by random mutagenesis of polyhydroxyalkanoate synthase. *Appl Microbiol Biotechnol* **102**, 7927-7934 (2018).
6. Kohlstedt M, *et al.* From lignin to nylon: Cascaded chemical and biochemical conversion using metabolically engineered *Pseudomonas putida*. *Metab Eng* **47**, 279-293 (2018).
7. Trudel P, Provost S, Massie B, Chartrand P, Wall L. pGATA: a positive

selection vector based on the toxicity of the transcription factor GATA-1 to bacteria. *Biotechniques* **20**, 684-693 (1996).
