# Supplementary material for: Development of a CRISPR/Cas9n-based tool for metabolic engineering of Pseudomonas putida for ferulic acid-to-polyhydroxyalkanoate bioconversion
Source: Commun Biol. 2020 Mar 5;3:98. doi: 10.1038/s42003-020-0824-5 (PMC7058019; doi:10.1038/s42003-020-0824-5)
Supplement: Supplementary file 2 — Description of Additional Supplementary Files [file 42003_2020_824_MOESM2_ESM.pdf]

Description of additional supplementary files

Supplementary Data 1. Source data used for graphs shown in Figure 2.

Supplementary Data 2. Source data used for graphs shown in Figure 3.

Supplementary Data 3. Source data used for graphs shown in Figure 4.

Supplementary Data 4. Source data used for graphs shown in Figure 5.

Supplementary Data 5. Source data used for graphs shown in Supplementary Figures 1, 2, 3 and 4.
